# Orodispersible Film (ODF) Platform Based on Maltodextrin for Therapeutical Applications

**DOI:** 10.3390/pharmaceutics14102011

**Published:** 2022-09-22

**Authors:** Irma E. Cupone, Andrea Sansone, Fabio Marra, Andrea M. Giori, Emmanuele A. Jannini

**Affiliations:** 1 Ibsa Farmaceutici Italia, Cassina de’ Pecchi, 20051 Milan, Italy; 2 Chair of Endocrinology and Medical Sexology (ENDOSEX), Department of Systems Medicine, University of Rome Tor Vergata, 00133 Rome, Italy; 3 Ibsa Farmaceutici Italia, 26900 Lodi, Italy

**Keywords:** orodispersible, film, drug, maltodextrin, sildenafil, vitamin D, treatment, therapy

## Abstract

Orodispersible film (ODF) is a new dosage form that disperses rapidly in the mouth without water or swallowing. The main ingredient of an ODF is a polymer that can be both of natural or synthetic origin. Maltodextrin is a natural polymer, mainly used in pharmaceutical and nutraceutical fields. This review aims to examine the literature regarding ODFs based on maltodextrin as the platform for developing new products for therapeutical application. ODFs based on maltodextrin contain plasticizers that enhance their flexibility and reduce their brittleness. Surfactants; fillers, such as homopolymer and copolymer of vinylacetate; flavour and sweetener were introduced to improve ODF characteristics. Both water-soluble and insoluble APIs were introduced up to 100 mg per dosage unit. The solvent casting method and hot-melt extrusion are the most useful techniques for preparing ODFs. In particular, the solvent casting method allows manufacturing processes to be developed from a lab scale to an industrial scale. ODFs based on maltodextrin are characterized in terms of mechanical properties, dissolution rate, taste and stability. ODFs made of maltodextrin, developed by IBSA, were tested in vivo to evaluate their bioequivalence and efficacy and were demonstrated to be a valid alternative to the marketed oral dosage forms.

## 1. Introduction

Orodispersible film (ODF) is an innovative oral dosage form whose presence on the pharmaceutical market is currently growing. ODFs first became a part of the seventh Pharmacopoeia European edition in 2012 [1]. According to this document (Ph. Eur.), ODFs are defined as single or multilayered sheets of suitable materials to be placed in the mouth where they disperse rapidly.

Pfizer introduced Listerine^®^ Pocket Packs thin strips in 2001. In 2003, the first drug-loaded oral film, Chloraseptic^®^ Relief Strips (OTC), was put on the market. The first prescription drug containing ODF (Ondansetron Rapidfilm) was approved in Europe in 2010 [2]. Vitamins [3], nutraceuticals, homeopathic remedies, and a number of other lifestyle products have been commercialized as ODFs [4,5].

The reasons for the success of new orodispersible films rely on the expected benefits for patients as well as for pharmaceutical companies. The interest of pharmaceutical companies in these innovative dosage forms is increasing more and more for several reasons: (i) because they may meet patient needs, (ii) because they can extend patent protection of branded Active Pharmaceutical Ingredients (APIs), (iii) because they can extend their product portfolio, and (iv) because ODFs can be registered easily as line extensions. Indeed, the oral route is the most preferred for the general population; it is easier, non-invasive, flexible and convenient particularly for the estimated 20% of the population that have psychological or physiological impairment to swallowing tablets or capsules [6]. Dysphagia affects 16–23% of the general population, increasing to 27% in adults over 76 years old. In fact, a number of older people experience swallowing problems [7]. In the presence of neurological disease (dementia, Parkinson’s disease, stroke), the prevalence of this difficulty is higher than in the general population. Eight out of ten patients prefer orodispersible dosage forms over conventional solid oral dosages [8]. Moreover, ODFs can be a suitable dosage form for children who are unable to swallow or have difficulties with swallowing tablets or capsules [9,10]. Finally, ODFs can also be useful for veterinary use [11]. Table 1 summarises the advantages of ODFs compared to other oral dosage forms.

The major disadvantages of ODFs are limited drug loading capacity, possible unpleasant taste of certain active ingredients, sensitivity to moisture and the higher cost of the manufacturing process compared to conventional products.

The main production methods for ODFs are the solvent casting method and hot-melt extrusion [17]. Solvent casting consists of preparing a liquid mixture that is spread on a liner and dried in an oven to evaporate the solvent. Hot-melt extrusion is a solvent-free manufacturing process in which the API is mixed with the excipients in solid state and then heated and pressed through a nozzle to deposit a layer, which is cooled down and cut to film size [14]. Other proposed methods are electrospinning or printing technologies [18].

A typical ODF consists of several technological elements: (i) drug or active substance; (ii) a film-forming polymer; (iii) a plasticizer agent to improve mechanical properties; (iv) a filler to improve toughness and structure; (v) saliva-stimulating agents to provoke salivation and facilitate disintegration; (vi) taste-masking agents such as flavours and sweeteners to cover the bitter and unpleasant taste of many APIs; (vii) colouring agents to make the film more attractive to consumers, and (viii) others such as surfactants, enzyme inhibitors, antioxidants, preservatives and thickening agents. Table 2 presents a summary of the most widely used excipients in ODF preparation.

Polymers, which are the backbone of all formulations, can be natural or synthetic. Cellulose and derivatives (hydroxypropyl cellulose, hydroxypropyl methylcellulose), pullulan, starch and modified starch (such as maltodextrin) are generally used [19]. Maltodextrins (MDX) are natural, safe, cheap, non-toxic and non-irritant polymers widely used in both pharmaceutical and food applications. They are water-soluble film-forming polymers [26,27,28]. A key feature of maltodextrin is the absence of gluten which makes products containing maltodextrin safe for people with celiac disease. Unlike other natural polymers, such as gelatin or chitosan, maltodextrins are also suitable for a vegetarian and vegan diet. Pullulan is a natural polymer with good film-forming properties, but it is very expensive [20], so it is usually used in combination with other polymers [19].

This narrative review focuses on orodispersible films containing maltodextrin as the main film-forming polymer, examining, in particular, the industrial and biopharmaceutical application. Currently, ODFs have a solid and growing presence in the pharmaceutical market, even if only a few drugs are today approved. Indeed, while there is plenty of literature on the development and lab-scale manufacturing of many ODFs, process scale-up and industrial production are poorly investigated and still quite challenging. Large-scale dedicated plants are rare, and ODF characteristics, such as thin dimension and high hydrophilicity, require careful fine-tuning of process parameters. Since several ODFs made of maltodextrins have been developed, manufactured on an industrial scale, in vitro and in vivo tested and commercialized, here we aim to carefully examine the current literature and to present the most recent technical advancements of this innovative industrial solution to deliver drugs to patients.


**Focus of the review**
✓Description of the main components of ODF based on maltodextrins;✓Identification of critical quality attributes (CQA);✓Manufacturing process on lab scale and industrial scale;✓Clinical application of ODF based on MDX.


## 2. Orodispersible Films Based on Maltodextrin

### 2.1. Composition of ODFs Based on Maltodextrin

**Figure 1:**
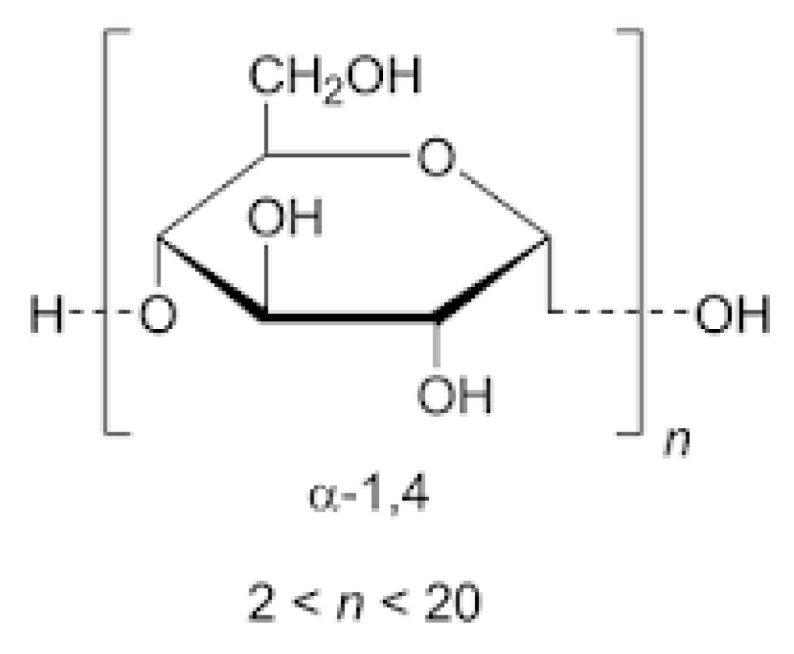
Maltodextrin: chemical structure and the main characteristics (n = glucose units per chain).

The essential excipients included in the ODFs are polymers, which are the backbone of film formulations, and plasticizers, usually adding up to 20% *w*/*w* [14]. Film-forming polymers are selected to allow the ODF to disintegrate rapidly in the oral cavity, to avoid stickiness and to assure suitable mechanical properties and stability at the same time. Maltodextrins (MDXs) (Figure 1) are largely used in pharmaceutical and food fields because are natural, safe and cheap polymers [26]. Both European and American pharmacopoeia present maltodextrin monographs.

MDX is a non-sweet nutritive saccharide mixture of polymers that consists of D-glucose units linked primarily by α-(1–4) bonds and with occasional α-(1–6) branches.

MDXs are prepared by the partial hydrolysis of a food-grade starch with suitable acids and/or enzymes. They are intermediate between starch and corn syrups, but unlike starch, they are soluble in cold water, and, unlike syrups, they are not sweet [29].

MDX digestion and metabolism are the result of salivary α-amilase in the mouth and, above all, of pancreatic α-amilase in the small intestine that breaks down MDX in a maltose unit. Maltase is responsible for the further metabolism that generates free glucose that undergoes the final absorption by the active transport of the enterocytes.

MDXs are freely soluble in water and slightly soluble to almost insoluble in alcohol [21]. MDX characteristics and classification are based on the parameter known as dextrose equivalence (DE). DE ranges from 2 to 20 and measures the reducing power of the sugars in the hydrolysate material. DE is an indicator of the degree of depolymerization of starch: the average molecular weight decreases as the DE value of the MDXs increases. Several physical and functional characteristics are affected by the DE value. Solubility, sweetness and hygroscopicity increase with increasing DE, whereas the viscosity, the anti-crystallizing power and the freezing temperature decrease as the DE increases. Low DE MDXs offer greater viscosity and improved film-forming properties than MDXs with higher DE [30]. MDXs are especially useful for delivering drugs because of their water solubility, low viscosity and high T_g_ after drying [31].

Many works in the literature describe the development of ODFs comprising a mixture of MDXs with other film-forming polymers [32,33], but the use of MDXs alone has scarcely been investigated. In this respect, Cilurzo et al. in 2008 [22] proposed a film made of MDX as the exclusive film-forming material. This technology is based on the patent EP1689347 “Self-supporting film for pharmaceutical and food use” [34]. According to this patented technology, MDXs constitute 40–80% of the ODF, and films made of MDX disintegrate within 1 min without leaving residues in the mouth, unlike hydrocolloids which tend to gel in contact with saliva

MDXs with different DE values were compared in ODFs. MDXs with a DE of 6 and 12 were compared in films containing nicotine [35], showing that decreasing the DE value of MDX improves the toughness of the film. Films containing benzydamine hydrochloride made of MDX with a lower DE value have better mechanical characteristics and lower water content [36].

Table 3 shows the main characteristics of MDX.

MDXs are the film-forming ingredients of the formulation, but it is necessary to add plasticizers and surfactants to improve the properties and the appearance of the ODFs.

Plasticizers enhance the flexibility and reduce the brittleness of orodispersible films by reducing the polymer glass transition temperature (T_g_). They help overcome brittleness after the drying process and allow the film to be punched and pouched in the primary packaging. Plasticizers reduce the intra-molecular interaction of the polymer by forming hydrogen bonds between the plasticizer and polymer, thus facilitating the movements of starch chains which result in greater flexibility. The addition of plasticizer reduces the elasticity of films and significantly increases the elongation at the break [37]. Low molecular weight polyols, such as glycerol, mannitol and sorbitol, are generally used as plasticizers [38]. Glycerol at 16–20% was found to produce ODFs containing MDX having a DE equal to 12 with suitable mechanical properties [22]. Plasticizers such as polyethylene glycol 400 (Peg400) and esters of citric acid are not recommended since they have low miscibility with MDX. Propylene glycol was discarded due to its unpleasant taste [22]. Amino acids were also proposed as non-conventional plasticizers [39]. Glycine and proline, in fact, can reduce the T_g_ of the MDX and improve the flexibility of the film obtained by mixing the polysaccharide with glycerine. In addition, residual water may also act as a plasticizer [3,40].

Surfactants lowering water tension are necessary to uniformly spread the water-based polymeric blend on the siliconized liner and to decrease the peel force required to separate the dried film from the release liner. Sorbitan oleate [35] was proposed as a surfactant. In the formulation of the vitamin D3 orodispersible film, a mixture of glyceryl monolinoleate:polysorbate 80, 75:25% *w*/*w* was used, with a calculated value of the hydrophilic–lipophilic balance (HLB) of 4.5 [3]. The suitable HLB value was selected to avoid excess foam during the mixing and to allow the water-based mass to be coated on the siliconized PET liner. Surfactants also play an important role in the enhancement of the dissolution of vitamin D3.

Films made of MDXs had a drawback concerning their physical stability, as they tend to harden over time. The addition of a homopolymer or copolymer of vinyl acetate avoided hardening of the films based on MDX and plasticizer. According to patent WO 2014/049548, copovidone and polyvinyl acetate were introduced in the MDX film formulation as fillers [41]. Franceschini et al. [42] demonstrated that by incorporating 3–5% of PVAc, the ODF made of maltodextrin was reinforced without affecting the dissolution rate of the ODF. Several products were developed on the basis of patents EP1689347, mentioned above, and this patent WO 2014/049548 as reported in Table 4, and these patents are the basis of the platform for ODFs applied by IBSA, a pharmaceutical company with dedicated plants for ODF industrial production.

Although oral films are not generally suitable for treatments requiring high dosages (i.e., >100–150 mg), the weight ratio API vs. excipients is more favorable than in most conventional dosage forms, reaching up to 50% *w*/*w* loading [48]. For example, an MDX-based sildenafil orodispersible film was loaded with up to 100 mg per dosage unit [43]. Drug dose can be modulated by modifying the width and thickness of the film.

Recently, Musazzi et al. demonstrated that ODFs made of MDX can be a suitable technological platform for loading lipid microparticles containing melatonin without significantly altering the shape and dimensions of the microparticles and the mechanical properties of the films [47]. Interestingly, the microparticles permitted efficacious modulation of melatonin release.

Drug substances loaded in the ODF do not necessarily have to be soluble in water. In fact, the active substance can be introduced in the matrix of films after a pre-solubilization or as a dispersion. For example, vitamin D3 has been successfully delivered through the MDXs as an oil solution [3].

**Figure 2:**
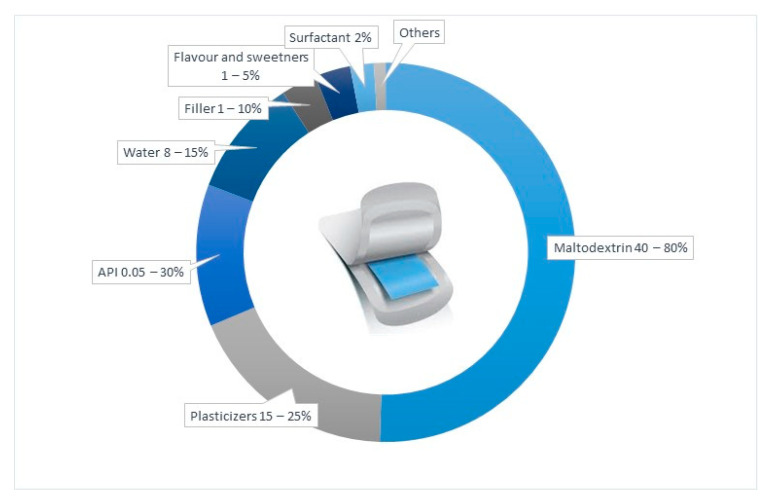
Typical composition of ODFs based on maltodextrins.

Other excipients can be used, in particular to meet organoleptic needs, such as the aforementioned taste-masking agents and sweeteners to cover the unpleasant taste of the majority of drug substances or a colouring agent to improve aspects of the ODF. Figure 2 shows a typical composition of a film based on maltodextrins, extrapolated from the examined literature.

### 2.2. Critical Quality Attributes

According to Ph. Eur., measures must be taken in the manufacture of ODF to ensure the suitable mechanical properties for ODFs and the appropriate drug release [49]. Therefore, the main mandatory technological tests reported in the Ph. Eur. are a dissolution test and tensile properties [20]. Moreover, the disintegration time is a critical parameter to evaluate during the development of the product because it can affect the bioavailability of the drug substance. Table 5 summarizes the specific requirements of ODFs according to the dedicated monograph of Ph. Eur.

The Eur. Ph. monograph on ODF refers to the dissolution test of the conventional solid dosage forms. Alongside the different formulation, ODFs made of maltodextrins were also investigated in the dissolution test. Vitamin D3 dissolved completely (>90%) within 30 min, but already after 15 min, the dissolved amount was greater than 85%, satisfying the requirements for the immediate release dosage forms [3]. In the case of a piroxicam (PRX) ODF, the dissolution rate of PRX was greatly influenced by the preparation method: films obtained by the casting method dissolved more rapidly than films obtained by hot-melt extrusion that contained microcrystalline cellulose (MCC). The different patterns recognized in the two groups were mainly ascribed to the presence of MCC in the formulation. After coming into contact with water, the film produced by hot-melt extrusion formed a swelled matrix. In the case of films obtained by the casting method, dissolution was completed after 10 min [22]. In vitro dissolution profiles of a sildenafil orodispersible film, obtained at three different buffers (pH 1.2, 4.5 and 5.5), were compared to that of the conventional film-coated tablet. The dissolution profiles of the sildenafil orodispersible film and the Viagra tablet overlapped. More than 85% of the label content dissolved within 15 min, so both drug products can be considered very rapidly dissolving [50], suggesting that the dissolution rate was mainly driven by the solubility of sildenafil in the media.

The European Pharmacopoeia specifies that ODFs should possess suitable mechanical strength to resist handling without being damaged. To evaluate mechanical properties, guidelines refer to the tensile properties of the thin plastic sheeting and foil materials used [51]. Mechanical properties are represented by tensile strength (TS), elongation at break (E%), elastic modulus or Young’s modulus (Y) and tensile energy to break (TEB). TS measures the toughness of the ODF, and it is calculated by dividing the maximum force by the original cross-sectional area of the specimen and expressed in force per unit area (MPa). E% is obtained by dividing the maximum extension of the film at the moment of rupture of the specimen by the initial gauge length of the specimen and multiplying by 100. Elastic modulus or Young’s modulus (Y) measures stiffness of the ODF. It is determined taking into account the slope of the linear portion of the stress–strain curve. The results are expressed in force per unit area (Mpa). Hard and brittle ODFs generally possess higher TS and higher Y values [52]. TEB is defined by the area under the stress–strain curve (AUC) per unit volume of the ODF. The TEB value reflects the toughness of ODF.

Some manufacturing steps, such as cutting, film formation and packaging, can be impacted by the mechanical properties of the film. Flexibility and plasticity of the film are crucial to allow the film to be handled without failure, but at the same time, the film should be tough enough to be self-supporting. Furthermore, elongation at break should be low to avoid deformation of films during the manufacturing process.

The main ingredient that promotes the suitable mechanical quality of the film is the film-forming polymers, while the amount of plasticizers modulate the mechanical properties. In the case of vitamin D3 ODF [3], glycerol is the main plasticizer, but residual water also acts as a plasticizer in the formulation. Water is the solvent used to solubilize maltodextrin, and after drying, a residual amount is required to confer suitable mechanical properties. The correlation between the water content and the mechanical properties of the film was investigated; TS decreased and E% increased proportionally to water content. In addition, in the case of a sildenafil ODF, the plasticizer activity of water was demonstrated; as the water content increases, the film becomes more elastic (E%) but less tough (TS). Moreover, it was demonstrated that the mechanical properties are not affected by the size of the specimen and the dosage strength; 25 and 100 mg sildenafil ODF were flexible and tough, and no significant differences were registered [53].

The effect of the presence of the drug in the ODF was investigated. The presence of piroxicam significantly modified the tensile properties of the film, leading to a relevant increase of stiffness. This result confirmed that the dispersion of a powder in the matrix of the film leads to an increase of the film fragility, even if the flexibility assay is still satisfactory [22]. In addition, the presence of diclofenac modified the mechanical properties of films, leading to a decrease of plasticity [44]. In contrast to diclofenac and piroxicam, the addition of nicotine slightly increased film plasticity. In particular, elastic modulus and tensile strength decreased, symptomatic of a lower stiffness [35].

Other important issues to take in consideration during ODF development are taste and stability.

Since ODFs disintegrate directly in the oral cavity, suitable taste is necessary. Many APIs have an unpleasant taste and different taste-masking techniques can be used, such as the addition of flavour and sweeteners, encapsulation or complexation with β-cyclodextrin or ion exchange resins. To evaluate the taste-masking efficiency during pharmaceutical formulation development, in vivo or in vitro methods have been proposed [44]. The in vivo studies included a human panel test. The palatability and the ease of use of the vitamin D3 ODF were evaluated [3]. In particular, the vitamin D3 ODF containing sucrose and orange flavour had a mostly likable taste of mild intensity, which left an aftertaste. The mouth feel was mainly judged as pleasant, and the use of the ODF was generally judged as very easy or easy.

In addition, in the case of a sildenafil ODF containing sucrose and lemon/grapefruit flavour as taste-masking agents, the majority of subjects (28/53; 52.8%) judged the palatability of the sildenafil 100 mg orodispersible film to be good/acceptable [43].

It is not always possible to evaluate the in vivo taste of a pharmaceutical product, both for ethical and toxicological reasons, especially when new drug substances are under investigation. Taste panels in children are nearly impossible, both for ethical reasons and for their inability to describe taste attributes [52]. Therefore, in vitro methods can be used.

The electronic tongue is a taste sensor with an array of multichannel detectors to mimic the conditions of taste buds in human tongues. The electronic tongue is a useful tool to taste active compounds in taste measurement technology. It is low cost and offers speed, simplicity and lack of risk. Diclofenac and nicotine were selected as model bitter drugs to evaluate the taste of ODF based on maltodextrin using the electronic tongue [35,44].

The stability studies of ODFs are performed according to the ICH guidelines for oral dosage forms [54]. In long-term, intermediate and accelerated stability studies, ODF properties are evaluated over time, and the compliance to the specifications is monitored.

Films based on maltodextrin were demonstrated to be chemically and microbiologically stable [3,45]. For a sildenafil ODF containing PVAc, the mechanical properties were evaluated in a stability study; at the end of the accelerated stability study (6 months), and during the intermediate and long-term stability study (24 months), tensile strength and elongation at break were calculated. Sildenafil orodispersible films were suitable to be handled for at least 6 months at 40 °C, for at least 24 months when stored at 25 and 30 °C and sufficiently flexible to be handled without failure. Restrained variation of the mechanical properties confirmed the stability of the film [53].

### 2.3. Manufacturing Process and Industrial Application

In general, the most commonly used techniques for preparing thin films are solvent casting and hot-melt extrusion. However, an innovative technique such as inkjet printing has evolved in the past few years [12,25].

The casting method consists of different steps. Generally, at first, a mass is prepared under temperature and stirring speed control. Afterwards, the mixture is spread and dried in a drying tunnel, controlling temperature, air circulation and coating speed once again. Then a slicing step follows, and in the last step, the films are punched, pouched and sealed in suitable single-dose sachets.

Hot-melt extrusion consists of the preparation of a mixture containing the drug, polymer and excipients melted and extruded to form a homogeneous and smooth film. This is a solvent-free process in which the use of the high temperature during extrusion causes a problem with the thermolabile substances [15]. The extrusion apparatus can be ram- or screw-based.

Cilurzo et al. demonstrated the possible application of screw-extrusion technology in the production of MDX ODF [22] and compared solvent casting and hot-melt extrusion for the preparation of placebo and piroxicam ODF. The casting method appeared more reliable to manufacture fast-dissolving films since the dosage forms exhibited the highest patient compliance and best performance in terms of in vivo and in vitro disintegration time. The granulation of MDX was necessary to properly feed the screw, and the addition of microcrystalline cellulose (MCC) was crucial in order to obtain smooth and non-sticking films. The film prepared by the casting method disintegrated in 10 s, while the film obtained by hot-melt extrusion disintegrated in 45 s. This difference was mainly attributed to the presence of MCC; the swelling of MCC retarded MDX dissolution. The dissolution rate of PRX was greatly influenced by the preparation method. These data indicated that the ingredients of the film affect the dissolution rate of the active ingredient.

**Figure 3:**
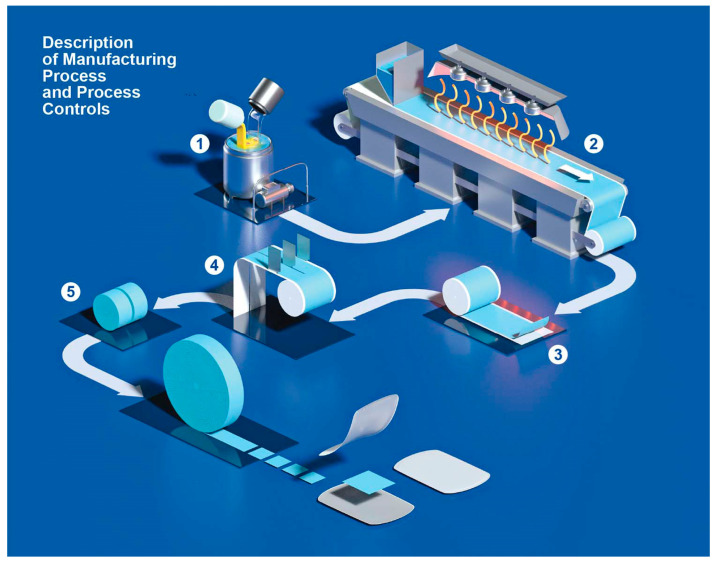
Description of industrial manufacturing process of ODF. The solvent-casting method, used to manufacture orodispersible films, consists of several steps. 1: During the first step, a liquid mixture is prepared; temperature and stirring speed are the critical process parameters. 2: Afterwards, the mixture is spread and dried in a drying tunnel by controlling temperature, air circulation, humidity and coating speed. 3: The dried and spread mixture is wound to form a big roll, called a Jumbo roll. 4: The jumbo roll is unwound to undergo two consecutive cutting phases, where the size of the film strips is selected and the final dosage is defined. 5: In the last step, the films are punched, pouched and sealed in suitable single-dose sachets.

Laboratory-scale ODFs can be cast into petri dishes or onto intermediate liners. For casting the film onto intermediate liners, different continuous and non-continuous manufacturing machines are available [55]. Solvent casting can be adapted to the production of small batches of ODF [18], but it is more suitable for an industrial manufacturing process. The scale-up from the bench scale to production scale is one of the biggest challenges. The manufacturing process of ODF made of MDX was successfully transferred from laboratory scale to industrial scale, and the feasibility of industrial manufacturing of ODF by the casting method was demonstrated [3]. Figure 3 shows in detail the steps of the OFD manufacturing process. The mixture, homogeneous and without bubbles and clumps, is spread on a support, and a siliconized PET sheet moves along the oven that at the end is coiled up onto a jumbo roll. The temperature and speed are set to remove excess water and to obtain a film with a homogeneous and smooth surface. The sizes and the strengths of the orodispersible films are defined by the consecutive process of slicing and formation. During film formation, the support is removed. Each orodispersible film is individually inserted between two packaging foils that are sealed by two sealing stations. A rotary knife cuts the single packaging unit after sealing. The variable data are printed on the sachet in line [3].

**Figure 4:**
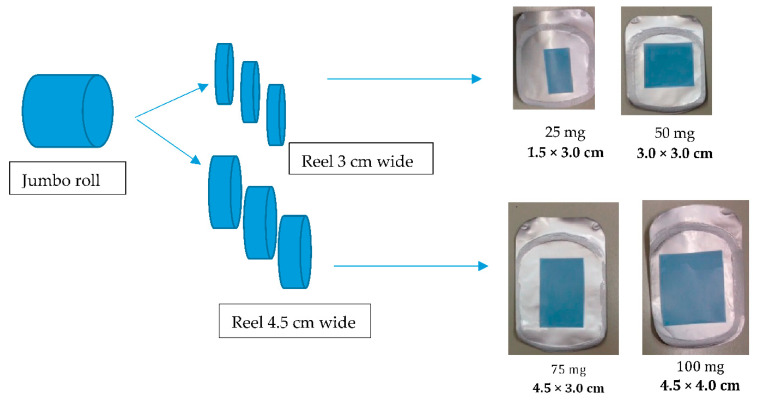
Manufacturing of orodispersible films of varying size. The jumbo roll is cut in reels of two different widths. Each reel is cut in films of two different dimensions.

Solvent casting is a very flexible process because various strengths can be obtained from the same jumbo roll using different film sizes. Figure 4 shows how the four different dosages of sildenafil citrate ODFs originate from only one mass, coated and dried in a jumbo roll. The slicing step produces reels of two widths (30 and 45 mm), defining the first dimension. In the last step, the reels are cut to form orodispersible films in four different sizes that determine the four different dosages [50,53].

The literature reports several critical factors for the solvent casting method: segregation or sedimentation in the coating mass, ripple effect, control of residual solvent and formation of air bubbles [56]. The study of the manufacturing process and a suitable scale-up of the process allowed these problems to be avoided. In the case of vitamin D3 ODF, the mixture obtained was homogeneous and without bubbles and clumps. The temperature of the ovens set between 90–110 °C and the coating speed set at 0.5 m/min provided suitable settings to have an industrial continuous process able to manufacture a homogeneous and smooth film, with a weight of 167 g/m^2^ and a thickness in the range 125 μm ± 5% [3].

Taking into account that about 40% of the drugs with market approval are poorly water soluble, the use of organic solvent during formulation development can be indispensable [56]. For instance, even if vitamin D3 is a poorly water-soluble substance, the use of organic solvent to obtain a homogeneous mixture was not necessary, with advantages in terms of the safety of both the manufacturing process and the finished product [3].

The most critical parameters for the scale-up of a product are the viscosity of the coating solution and the drying temperature [55]. It was demonstrated that the suitable viscosity value has to be in the range 2000–5000 cP. The vitamin D3 mass was formulated to have a viscosity of about 3500 cP; this value is suitable for the industrial coating process and is the result of a mixture containing 39.0% water [3].

Other kinds of technologies were proposed to obtain orodispersible films made of MDX such as hot-melt ram extrusion 3D printing [57]. This technology consists of a simple procedure involving mixing the active ingredient with maltodextrin and other excipients. Hot-melt printing was proposed to prepare palatable ODF loaded with diclofenac. This technology could be used advantageously in the hospital pharmacy setting to allow precise personalization of the dose [58].

### 2.4. Clinical Application

Orodispersible films are a valid solution to improve patient compliance and medication adherence. The lack of compliance related to the intake of solid dosage forms is relevant in patients who have difficulty swallowing tablets or capsules, such as paediatrics and geriatrics [9,59]. The acceptability of ODFs by infants and preschool children was also evaluated. Children and their caregivers showed a high degree of acceptance, and ODFs were shown to be an age-appropriate dosage form for infants and preschool children [13]. Ninety percent of paediatric formulations are in liquid form since they are easier to swallow for children and easy to adjust the dose to the weight of the child [60]. Superiority in acceptance and swallowability of an ODF compared to syrup was demonstrated, and the palatability assessments were in favour of the ODF [61].

Contrary to expectations, the use of an ODF instead of a conventional immediate release dosage form does not generally affect the bioavailability of the API [62]. It is generally perceived that ODF provides a faster onset of action than conventional immediate release tablets or capsules thanks to a more rapid absorption through oral mucosa. However, the residence time of the drug on the pre-gastric mucosae is quite short. The literature reports only a few studies of improved bioavailability of API in orodispersible forms, such as selegiline, flupentixol, ketoprofen and meloxicam [6].

Bioequivalence of ODFs made of maltodextrins with conventional dosage forms was evaluated with two different APIs: sildenafil citrate and vitamin D3.

Sildenafil was the first selective inhibitor of cGMP-specific type 5 phosphodiesterase (PDE5), localized in the smooth muscle cells of the corpora cavernosa [63], available on the market as oral therapy for erectile dysfunction (ED). Sildenafil is a safe, effective and well-tolerated oral agent for the treatment of ED and is predominantly metabolized by cytochrome P-450 into an N-desmethyl metabolite that accounts for approximately one-fifth of the drug’s activity [64]. Interestingly, a comparison between the PDE5 inhibitors (PDE5i) showed that sildenafil expresses a particular ability to ameliorate penile performance as measured by penile colour Doppler ultrasonography in a spontaneous, open-label, randomized, multicenter, crossover study [65]. Notably, the best efficacy on some of the penile flow parameters within the 8-week treatment period were seen with sildenafil 100 mg, and with lower strength with sildenafil 50 mg. Future research should concentrate on intermediate strengths.

A novel sildenafil orodispersible film made of maltodextrin containing sildenafil citrate has recently been marketed. The ODFs are available for the first time in four different dosage forms (25, 50, 75 and 100 mg). Radicioni [43] described the bioequivalence study between the new sildenafil 100 mg orodispersible film and the conventional marketed 100 mg film-coated tablet after a single dose administration. Secondary outcomes were also monitored; all volunteers confirmed the complete disintegration within 1 min after administration, and the majority of subjects judged the palatability of the sildenafil 100 mg orodispersible film to be good/acceptable. The bioequivalence test was fully satisfied for sildenafil and N-desmethyl-sildenafil in terms of rate and extent of bioavailability.

Cocci [66] compared 75 mg orodispersible films made of maltodextrin and 100 mg film-coated tablets, evaluating efficacy and safety. Interestingly, the new dose of 75 mg, intermediate between the most popular (50 mg) and the most powerful (100 mg), is available for the first time [67]. The median action time of sildenafil in this study was 20 min. Both the new and the highest formulations were safe and effective, and no additional side effects arose for the ODF formulation, thus suggesting that oral film can be used interchangeably with the conventional oral forms, having all the characteristics of the conventional formulations plus the special features of the new ODF form.

In particular, it has been demonstrated in a number of real-life surveys that PDE5i in the traditional forms do not meet all the expectancies and needs of the patient [68,69]. *Viagra Jealousy* is a figure-of-speech created for communication with the patient which encompasses the typical reactions of the partner, usually female, against the use of any kind of PDE5i (Table 6).

It appears, in fact, that a number of women may strongly oppose the use of PDE-5i because they view their partner’s use of these drugs as meaning that they have lost their sex appeal. They may also think medications are potentially harmful for their partner in general and for cardiovascular health in particular. Moreover, this may represent a dramatic limiting factor and reason for abstinence, coupled with the stigma of being treated for ED, which is still a major issue for many patients [70]. This may express the potential to improve treatment adherence, thereby enhancing the sexual health and sense of psychological well-being of patients [71].

For all reasons, a formulation such as ODF, conveniently carried in the wallet and not in the traditional pill box, consumed without water, rapidly disintegrating and without the appearance of the traditional pill (which is, in the case of sildenafil, a really popular icon) could genuinely be considered a brilliant solution to respect the need for discreet treatment many patients may have [72].

Sildenafil pharmacokinetics associated with sublingual administration of either ODF or an orodispersible tablet in comparison to the film-coated tablet as the original formulation was evaluated by De Toni et al. who investigated the release and permeation profile of the different sildenafil formulations in in vitro systems specifically developed to evaluate the transmucosal absorption of drugs [68].

Transmucosal absorption was excluded by Loprete [73] who investigated the bioavailability of sildenafil after sublingual and supralingual ODF administration. The data obtained showed that the absorption of sildenafil through oral mucosa was insignificant and did not significantly affect the drug kinetics. After the complete dissolution of the ODF in the mouth, sildenafil was likely swallowed and absorbed further down in the gastrointestinal tract, regardless of the place of disintegration of the film. Sildenafil film was safely administered both sublingually and supralingually.

At the moment, the literature reports conflicting evidence on the mucosal absorption of sildenafil after ODF intake. The topic is still a subject of study and should be further evaluated in future studies.

Another dramatically important advantage could be identified in a sildenafil ODF. PDE5is are the most widespread counterfeited drugs, with an enormous worldwide market. The counterfeit PDE5is may carry substantial risks for the physical and psychological heath of the buyers [74,75]. So far, strategies to face counterfeits (education of clinicians and pharmacists, involvement of international authorities, social media interventions) have substantially failed to meet their aim [76]. The relatively expensive, high-tech ODF formulation described here may represent a unique strategy for the purpose of countering the spread of counterfeits, as demonstrated by the apparent absence of this formulation on the black market [77].

IBSA developed an ODF based on maltodextrin and containing 25,000 IU of vitamin D to rapidly dissolve and/or disintegrate when placed in the mouth without drinking and chewing.

Vitamin D3 has long been known for its influence on bone health by regulating calcium homeostasis and bone metabolism. Vitamin D deficiency in adults is responsible for osteomalacia that provokes a poorly mineralized skeletal matrix, and in the elderly is the cause of osteoporosis and muscle weakness, two factors that induce fractures after falls in the elderly [78].

Recently, the role of vitamin D3 as a regulator of the immune system was investigated. Vitamin D’s role as an important stimulant for innate immunity and support of the adaptive immune system was highlighted [79]. Vitamin D3 acts as an important mediator of innate immune responses, enhancing the antimicrobial activity of immune cells such as monocytes and macrophages [80].

Recent studies recognize the direct antiviral activity of vitamin D that inhibits virus replication. Vitamin D also plays a role in inflammatory response, modulating the relative concentration of anti-inflammatory cytokines and pro-inflammatory cytokines. Vitamin D3 prevents the damage of pneumonia by reducing the concentration of pro-inflammatory cytokines in lung tissue [81]. Studies reported that daily or weekly consumption of vitamin D3 by people affected by vitamin D deficiency can protect from acute respiratory infection. It also showed protective effects against acute respiratory tract infection especially in persons with vitamin D deficiency. Vitamin D3 is associated with influenza and COVID-19, with serious and deadly consequences [82].

Forty-eight subjects were randomized to receive a single dose of vitamin D3 25,000 IU ODF made of maltodextrin under fasting conditions, a single dose of vitamin D3 ODF under fed conditions or a single dose of vitamin D3 25,000 IU oral solution [45]. The results support the conclusion that the bioavailability of exogenous calcifediol following a single 25,000 IU ODF intake is comparable to or better than an equivalent dose of the reference oral solution when administered under the same (fed) conditions in terms of both rate (Cmax) and extent (AUC0-t = 72 h) of absorption. Although the AUC0-t and AUC0-∞ of the exogenous 25(OH)D3 tended to be slightly higher under fed conditions, indicating that the intake of a meal within 30 min of dosing can slightly enhance the extent of absorption, from a clinical viewpoint, the extent of food effect on the bioavailability of the ODF can be considered negligible or absent.

Moreover, the ODF containing vitamin D3 25,000 IU had a likable taste of mild intensity, which left an aftertaste. The mouth feel was mainly judged as pleasant, and the use of the ODF was generally judged as very easy or easy. The ODF provided a valuable alternative to the marketed oral solution.

## 3. Conclusions

ODFs based on maltodextrin were found to be a flexible platform to formulate pharmaceutical product without organic solvent, containing drug substances with different characteristics, both water-soluble and insoluble, and in a very wide dosage strength range up to 100 mg (Sildenafil ODF IBSA FilmTec^TM^). ODFs made of maltodextrin were characterized by excellent mechanical properties, chemical and physical stability and good taste. They can be produced on an industrial scale with several processes and can be a valid alternative to conventional dosage forms for safety, efficacy and bioequivalence.

Future perspectives could be the use of this platform for further pharmaceutical and nutraceutical development for application in particular categories of patients such as paediatric patients, non-cooperative patients, dysphagic patients or for veterinary use. The advantages of the use of MDX to prepare ODFs are the high water solubility, the extremely safe and non-toxic nature and the rapid disintegration of the polymer without swelling and residual fragments in the mouth. The manufacturing process of ODF requires special precautions, and only pharmaceutical industries focused on this product can develop and manufacture ODF with the suitable properties and compliant to the required CQAs. In the future, the manufacturing process on an industrial scale can be increased to obtain a standard manufacturing process for routine manufacture.

Even though there is a growing interest in orodispersible films, at the moment there are few products on the market. The optimization of manufacturing processes and definition of a standard method for characterization could help increase commercial availability.

## Figures and Tables

**Table 1:** Advantages of orodispersible films (ODF) in comparison to traditional oral dosage forms.

Advantages
Dissolve rapidly in the saliva
They do not have to be swallowed
Suitable for paediatric and geriatric patients [12,13]
Suitable for disabled patients, dysphagic patients, patients suffering from Parkinson disease, mucositis or nausea [14]
Ideal for people who do not have continuous access to water [15]
Flexible but still robust compared to orodispersible tablets [16]
Accurate dosing compared with syrup or drops
Improved bioavailability because of a potential oromucosal absorption
Improve therapy adherence in patients [6]

**Table 2:** Typical excipients used in orodispersible films (ODF) preparation.

Excipients		Examples of Excipients	References
Polymer	Natural	pullulan, gelatin, maltodextrin, hydroxypropyl methyl cellulose, chitosan, sodium alginate	[12,16,19,20,21]
	Synthetic	carboxymethyl cellulose, hydroxypropyl cellulose, polyvinyl pyrrolidone	
Plasticizers		glycerin, sorbitol, mannitol, propylene glycol, triacetin, citrate ether	[22,23]
Surfactants		sorbitan oleate, glyceril monolinoleate	[3]
Taste-masking agents		essential oil natural or synthetic flavours, cyclodextrin	[24]
Other: Sweeteners Color Saliva stimulators		sucrose, fructose sucralose, saccharin, cyclamate, acesulfame K titanium dioxide citric acid, malic acid, tartaric acid	[25]

**Table 3:** Main characteristics of maltodextrin.

- Good film former - Good solubility in water - Natural - Safe, non-toxic, non irritant - Cheap - Odourless and tastless - Gluten-free - Vegetarian and vegan use - USP-NF and Eur Ph complied

**Table 4:** Example of proposed ODF based on maltodextrin as the film-forming polymer.

API	Dosage Strength	Drug Amount (%)	Other Excipients	Reference
Nicotine	0.5 mg	0.5%	glycerol, sorbitan oleate, mint and milk flavour, citric acid	[35]
Sildenafil citrate (IBSA FilmTec^TM^)	25 mg	26%	maltodextrin, sucralose, lemon flavour, grapefruit flavour, polyvinyl acetate dispersion 30%, titanium dioxide, indigotine E132, polysorbate 20, propylene glycol monocaprylate, glycerol, water	[43]
	50 mg			
	75 mg			
	100 mg			
Diclofenac sodium	25 mg as diclofenac	13.4%	glycerol, sorbitan oleate, sucralose, mint and liquorice flavours.	[44]
Vitamin D3 food supplement (IBSA FilmTec^TM^)	1000 IU 2000 IU	0.06%	maltodextrin, glycerol, mannitol, extra virgin oil, orange flavour, copovidone, vitamin C, vitamin E, glycerol monolinoleate, polysorbate 80, sucralose, titanium dioxide, iron oxide, alginate	[3]
Vitamin D3 Pharmaceutical (IBSA FilmTec^TM^)	25,000 IU 50,000 IU	0.5%	maltodextrin, glycerol, mannitol, refined olive oil, orange flavour, copovidone, vitamin C, vitamin E, glycerol monolinoleate, polysorbate 80, sucralose, titanium dioxide, alginate	[45]
Piroxicam	25 mg	9–15%	maltodextrin, glycerol, sorbitan oleate	[20]
Olanzapine	9.4 mg 12.6 mg	5.1% 6.3%	maltodextrin, glycerol, sorbitan oleate	[46]
Melatonin	--	2.5–11.4%	maltodextrin, Span 80, Tween 80 and glycerol	[47]

**Table 5:** Requirements of ODFs according to Ph. Eur.

✓ Rapid dispersion in the mouth ✓ Suitable mechanical strength to resist handling without being damaged ✓ Appropriate release of the active substance.

**Table 6:** The Viagra Jealousy, i.e., the principal reasons, explicit or implicit, for female dissatisfaction with the use of any kind of type 5 phosphodiesterase inhibitor in its conventional formulations.

1. Reduction of personal sex appeal
2. Risks for extramarital affairs
3. Risks for general health
4. Risks for cardiovascular health
5. Risks for fertility

## Data Availability

Not applicable.
